# Antiproliferative effects, mechanism of action and tumor reduction studies in a lung cancer xenograft mouse model of an organometallic gold(i) alkynyl complex†

**DOI:** 10.1039/d4md00964a

**Published:** 2025-03-24

**Authors:** Uttara Basu, Anna Wilsmann, Sebastian Türck, Henrik Hoffmeister, Matthias Schiedel, Gilles Gasser, Ingo Ott

**Affiliations:** a Institute of Medicinal and Pharmaceutical Chemistry, Technische Universität Braunschweig Beethovenstr. 55 38106 Braunschweig Germany ingo.ott@tu-braunschweig.de; b Department of Chemistry, BITS Pilani K K Birla Goa Campus NH 17B Bypass Road Goa 403726 India uttarab@goa.bits-pilani.ac.in; c Chimie Paris Tech, PSL University, CNRS, Institute of Chemistry for Life and Health Sciences 75005 Paris France

## Abstract

Organometallic complexes offer a wide range of properties like structural variety, reaction kinetics, tunable lipophilicity and alternate mechanisms of activation under physiological conditions compared to platinum chemotherapeutics and are thus being explored for their potential anticancer applications. In this regard, gold(i) organometallics hold a pivotal position for their ability to act on biological targets different from DNA (which is the primary target of platinum therapeutics), such as thioredoxin reductase. Here, we report on the stability, *in vitro* antiproliferative effects, protein binding, cellular uptake, mechanism of action, effects on mitochondrial respiration of cancer cells as well as *in vivo* tolerance, toxicity and tumor reduction in an A549 lung cancer xenograft mouse model of an organometallic gold(i) complex (1) bearing 4-ethynylanisole and triethylphosphane as ligands. The complex, which was stable in DMSO and reactive towards *N*-acetylcysteine, triggered strong antiproliferative effects in various cancer cell lines and had a protein binding of approximately 65% that reduced its generally efficient uptake into tumor cells. Antimetastatic properties were indicated for 1 in a scratch assay and strong inhibition of thioredoxin reductase (TrxR) was confirmed for the purified enzyme as well as in A549 lung cancer cells, which strongly overexpress TrxR. Real time monitoring of the oxygen consumption rate in multiple cancer cell lines, using the Seahorse Mito stress assay, demonstrated that mitochondrial respiration was severely disrupted, showing a significantly low oxygen consumption rate. Other respiratory parameters, such as proton efflux, spare respiratory capacity and maximal respiration, were also attenuated upon treatment with 1. The complex was well tolerated *in vivo* in mice at a dose of 10 mg kg^−1^ and showed tumor reduction compared to the control group of animals in a lung cancer xenograft model of nude mice. In summary, complex 1 represents a novel organometallic anticancer drug candidate with a mechanism related to TrxR inhibition and mitochondrial respiration inhibition, showing efficient *in vivo* antitumor efficacy.

## Abstract 2

An organometallic anticancer drug candidate with a mechanism related to TrxR inhibition and mitochondrial respiration inhibition shows efficient *in vivo* antitumor efficacy.

## Graphical abstract

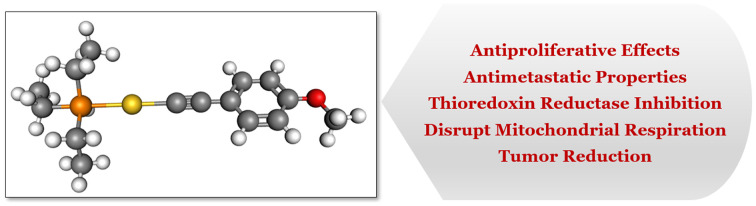

## 1. Introduction

The medicinal properties of gold have been known to humankind for several centuries when it was used to treat ulcers and microbial infections. Historically, Robert Koch's discovery of the toxicity of potassium dicyanoaurate (K[Au(CN)_2_]) to the tuberculosis bacillus *in vitro* indicated the great potential of gold complexes for the first time in modern medicine. Later, the introduction of gold compounds as therapeutics for rheumatoid arthritis by Jaques Forestier led to the development of antirheumatic gold drugs. This propelled research towards the discovery of novel gold complexes for various other therapeutic applications.^1–6^ Auranofin, aurothioglucose and aurothiomalate are the prominent gold-based, FDA approved antiarthritic drugs.^7,8^ Auranofin is also in clinical trials for ovarian cancer (NCT03456700, NCT01747798), chronic lymphocytic leukemia (NCT01419691), HIV (NCT02176135) and protozoal diseases (NCT02736968). It is well documented that the treatment with cisplatin, the classical platinum-containing chemotherapeutic drug, suffers from several limitations such as lack of specificity, development of cancer cell resistance and various side effects, and alternative candidates are necessary to combat cancer.^9^ Organometallic compounds are interesting as they are structurally diverse, can undergo various types of reactions by ligand exchange, redox pathways, *etc.* and may be explored for generating drug candidates for cancer therapy.^10–12^ Specifically, gold complexes have attracted the attention of scientists because of their *in vitro* anticancer properties towards platinum-resistant cell lines.^13,14^ In this regard, several gold compounds have been designed and synthesized with ligands like N-heterocyclic carbenes (NHCs), phosphines, thiolates, alkynes, BODIPY or heterobimetallic complexes, *etc.* They are being explored as potential chemotherapeutic agents with different modes of action compared to platinum drugs.^15–39^

Gold(i) has a higher affinity for soft ligands, like those with sulfur donor atoms, making thiol groups of enzymes, which are sometimes overexpressed in cancer cells, attractive molecular targets. Previous studies have indicated a multimodal action of gold complexes towards cancer cell lines, where thioredoxin reductase (TrxR), a selenocysteine containing enzyme that maintains the redox homeostasis system of the human physiology, has emerged as the most relevant target.^40–42^ Other enzymes, such as glutathione reductase, cysteine proteases, or protein tyrosine phosphatases, are also possible targets for gold complexes.^43,44^ It has also been reported that gold complexes prefer localizing in the mitochondria of cells and interfere with pathways associated with oxidative phosphorylation that drive cellular respiration. This is advantageous considering that an isoform of the thioredoxin reductase enzyme, TrxR2, is found inside the mitochondria of cells.

**Fig. 1:**
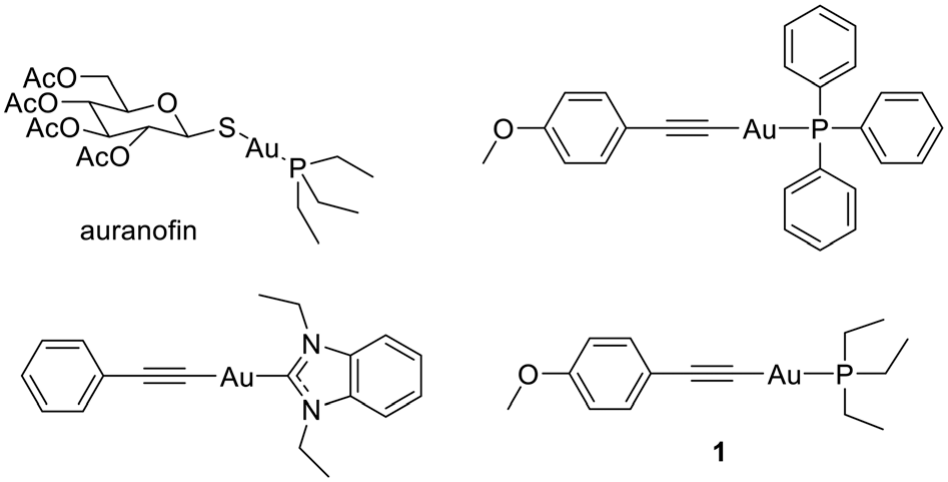
Structures of the FDA approved antiarthritic gold(i) drug auranofin and of reported organometallic gold(i) complexes bearing an alkynyl ligand, such as 1.^15,32^

We and others have recently reported on the promising anticancer properties of gold complexes with alkynyl ligands (see Fig. 1 for examples).^15,26,32,34,45,46^ These complexes have the general advantage of an increased stability due to the strong coordination of the alkynyl ligand to the gold center. Many of these complexes triggered strong effects against cancer cells. For some examples, additional important anticancer effects were reported, such as anti-angiogenic properties that were confirmed in a zebrafish model.^45^ In a previous study, we evaluated the influence of the type of phosphine ligand in complexes of the general type alkynyl-gold(i)(phosphine) and studied a promising example with a triphenylphosphine ligand *in vivo*.^32^ The derivative with the triphenylphosphine ligand, however, remained inactive *in vivo*, which could be attributed to the solubility issue related to the lipophilic nature of the triphenylphosphine structure. Structures with an improved solubility were suggested for further studies. Importantly, aggregation effects on the biological activity of gold alkynyl complexes have been recently highlighted by Rodríguez and coworkers.^23^

Building on these previous findings, we selected complex 1 (Fig. 1) with the less lipophilic triethylphosphine ligand for further studies as a potential anticancer agent. Computational studies have indicated the high stability of 1. Importantly, it was found to trigger strong antiproliferative effects and to strongly inhibit TrxR, making it an ideal candidate for further development as an anticancer lead compound.^32^ Here, we report about the *in vitro* antiproliferative properties in different cell lines, mechanistic aspects, and *in vivo* behavior in an A549 cell line-derived tumor xenograft (CDX) subcutaneous model.

## 2. Results and discussion

### 2.1 Chemistry

Complex 1 was synthesized with high purity and good yield using a one-step reaction between chloro(triethylphosphine)gold(i) and 4-ethynylanisole in a mixture of methanol and dichloromethane. The product was isolated as a yellow precipitate and dried in a vacuum. The method described in the Materials and methods section is based on our previous reported procedure with modifications to improve the yield.^32^ The structures and the bond dissociation energies of complex 1 have been reported using DFT calculations by our group recently and indicated high stability.^32^ According to our observations, 1 was very stable at room temperature in the solid state and could be stored for several months without any changes in its composition.

### 2.2 Stability in solution and in the presence of *N*-acetylcysteine

**Fig. 2:**
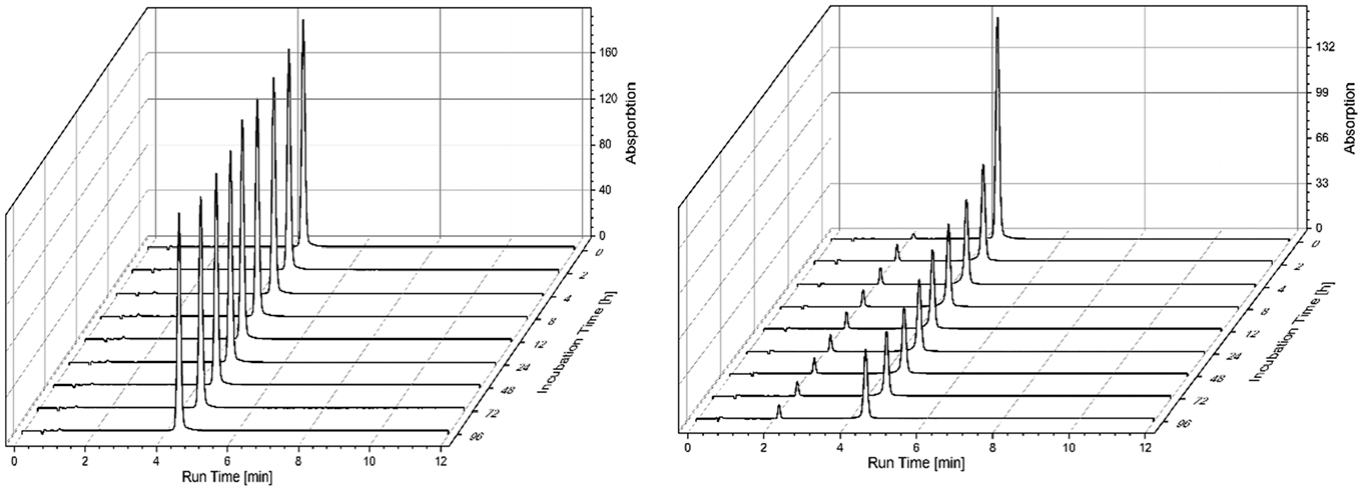
HPLC-MS analysis of 400 μM 1 in DMSO (left) and a 400 μM equimolar mixture of 1 and NAC at 37 °C over 96 h; retention times: 4.5 min for 1 and 2.2 min for 4-ethynylanisole.

DMSO is the most frequently used solvent for preparing stock solutions of compounds for biological assays. However, this solvent also has coordinative properties that can influence the stability of metal complexes.^47,48^ To evaluate the stability of 1 in DMSO, a 400 μM solution was kept at 37 °C and analyzed by HPLC over a period of 4 days (Fig. 2). Complex 1 showed excellent stability in DMSO with a recovery rate of 97% after 96 h, confirming that DMSO can be used without problems for preparing stock solutions of 1 and that the complex is stable in a polar organic solvent under physiological conditions. Next, the stability of 1 in the presence of *N*-acetylcysteine (NAC) was checked by analysis of an equimolar mixture of 1 and NAC in DMSO/phosphate buffered saline (PBS) 1/1 (v/v) under the same conditions. As a thiol-containing biomolecule, NAC can react with gold-containing compounds under ligand exchange reactions. As expected, there was an immediate reaction between NAC and 1 with 88% of 1 being intact at the beginning of the experiment (0 h). After 2 h, the recovery rate for 1 was 45% and slowly decreased to 31% after 96 h. The release of the free alkyne ligand could be further confirmed by a spiking experiment. These results are overall in good agreement with 1 being a “thiol-reactive” probe and TrxR inhibitor.

### 2.3 Antiproliferative effects

The efficacy of 1 as an antiproliferative agent was evaluated using a cell viability assay in a panel of different cancer cell lines, consisting of breast cancer MCF-7, triple negative breast cancer MDA-MB-231, colon cancer HT-29, lung cancer A549 cells and non-tumorigenic kidney RC-124 cells. Auranofin (AF) was used as a reference compound. It was found that 1 was strongly antiproliferative in all the cell lines, with IC_50_ values between 2 and 12 μM, similar to those of auranofin (Table 1). The activity of 1 against HT-29 and MDA-MB-231 cells has already been studied in our previous report and yielded IC_50_ values of 2.6 μM and 1.1 μM, respectively.^32^ The slightly higher activity in our previous report could be due to differences in the experimental protocols, such as a fresh batch of cells in the current study and a different seeding density. It has to be noted that the related chloride complex, (triethylphosphine)AuCl, has been reported to have high cytotoxicity to various colon carcinoma cell lines including HT-29 with an IC_50_ value near 1 μM,^49^ while the alkynyl ligand 4-ethynylanisole showed negligible cytotoxicity under similar experimental conditions. Both auranofin and 1 were active against the non-tumor cell line RC-124, which is in line with our previous results obtained with this cell line.^28^

**Table 1:** Antiproliferative effects of complex 1 and auranofin in different cell lines given as IC_50_ concentrations in micromolar (μM) units

Compound	HT-29	MDA-MB-231	MCF-7	A549	RC-124
IC_50_ values (μM)					
1 (4-MeO-Ph-≡-Au-PEt_3_)	6.8 ± 0.2	2.3 ± 0.5	2.9 ± 0.3	12.1 ± 1.4	3.1 ± 0.8
Auranofin (AF)	3.2 ± 0.4	1.9 ± 0.6	1.4 ± 0.2	8.9 ± 2.6	2.0 ± 1.2
4-Ethynylanisole	>50	>50	45.6 ± 1.8	>50	>50

### 2.4 Protein binding and cellular uptake

Binding of drugs to proteins, especially to plasma proteins like albumin, has enormous effects on the *in vitro* cellular uptake and *in vivo* biodistribution. While binding to proteins can reduce the cellular uptake, in the case of reversible binding, the conjugate can serve as a reservoir of the drug and allow slow and controlled drug release leading to a better biodistribution profile.^50^ The binding to albumin reduces the concentration of the free drug and results in the formation of a larger complex that has a lower diffusion rate across the cell membrane. Therefore, high protein binding can strongly reduce the bioactivity of drugs in cell-based experiments.

**Fig. 3:**
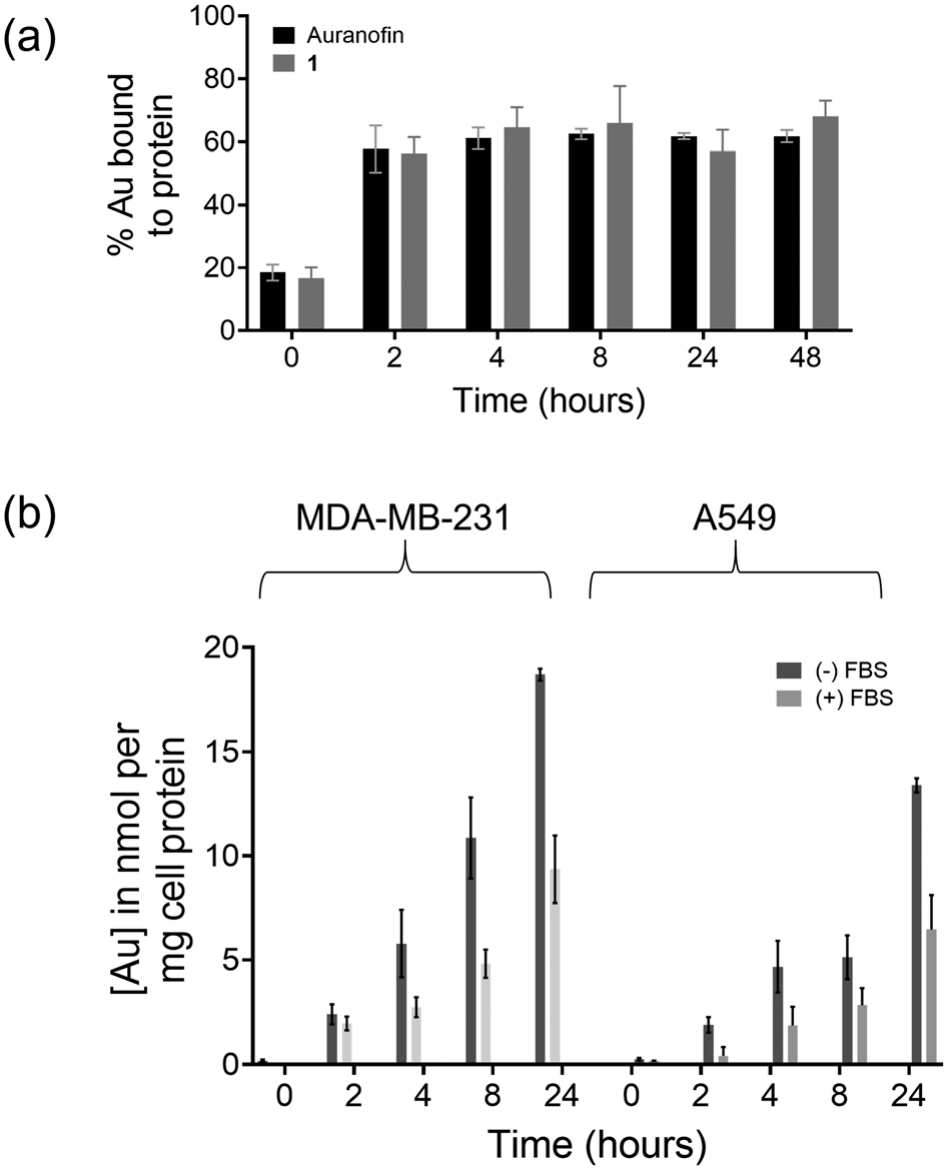
(a) Percentage of gold bound to proteins in the presence of fetal bovine serum (FBS) after incubation at different time points measured using ICP-OES; (b) cellular uptake of complex 1 in MDA-MB-231 and A549 cells after different incubation times in the presence or absence of FBS.

To check the protein binding ability of 1, we performed a protein binding assay with a fetal bovine serum (FBS) medium, which contains important serum proteins (Fig. 3). Auranofin was used under identical experimental conditions as the reference compound. The respective complexes were added to the medium, and aliquots were collected at different time points. Ethanol was added to precipitate the proteins and the unbound gold content was determined in the supernatant using inductively coupled plasma optical emission spectroscopy (ICP-OES). The metal bound to FBS proteins was calculated (%) and the results are depicted in Fig. 3. Both auranofin and 1 had a fast and efficient binding that reached a plateau within 2 h of incubation, where approx. 56–68% of gold was bound to proteins. There was no significant difference between 1 and auranofin in this assay. High protein binding was also observed with gold alkynyl complexes with N-heterocyclic carbene ligands in our recent work.^15^

It has been demonstrated that a higher accumulation of metallodrugs generally potentiates antiproliferative activity. Moreover, bioactivity of antitumor metallodrugs is influenced by a multi-step process that includes, as the first step, cell entry or accumulation. The cellular uptake of any potential chemotherapeutic agent is thus of paramount importance and many inorganic complexes are not able to cross the cell membrane due to various factors, such as low aqueous solubility, high charge, low lipophilicity and inactivation by covalent binding to serum proteins. We thus studied the time-dependent uptake of 1 into MDA-MB-231 and A549 cells with and without FBS using atomic absorption spectrophotometry. Cells were incubated with 2.0 μM and 5.0 μM of complex 1 for MDA-MB-231 and A549 cells, respectively, and the gold content was measured in the cell lysate after different time points up to 48 h. The obtained values of up to 20 nmol gold per milligram cell protein were roughly within the same order of magnitude as with different gold alkynyl complexes studied under comparable experimental conditions.^26,36,37,39,45,46^ Different concentrations were used because of the differences in the IC_50_ values in the two cell lines and shorter treatment times were chosen to allow the detection without compromising cell viability. In all cases, it was evident that the uptake was higher when using a serum-free cell culture medium. After 2 h of incubation in FBS free medium, there was a marginal increase in the cellular gold levels with the values being nearly the same for both cell lines. At 4 h, the amount of metal in cells was 4–5 times higher than in untreated samples, which nearly doubled after 8 h in MDA-MB-231 cells; however, it increased only marginally for A549 cells. The value further increased nearly 15–20 times after 24 h of incubation compared to the control samples for both A549 and MDA-MB-231 cells, respectively. This is in stark contrast to experiments conducted in the presence of serum where the concentration of gold found in cell lysates was fairly modest until 4 h in both cell lines and showed a moderate increase slightly after 8 h of incubation with 1. Even then, the amount of gold compared to the FBS free samples was about half the value. Moreover, cell uptake was marginally faster in MDA-MB-231 cells compared to A549 cells. In summary, the results clearly show that the presence of serum reduces the cellular uptake of 1 (Fig. 3).

### 2.5 Live cell imaging

**Fig. 4:**
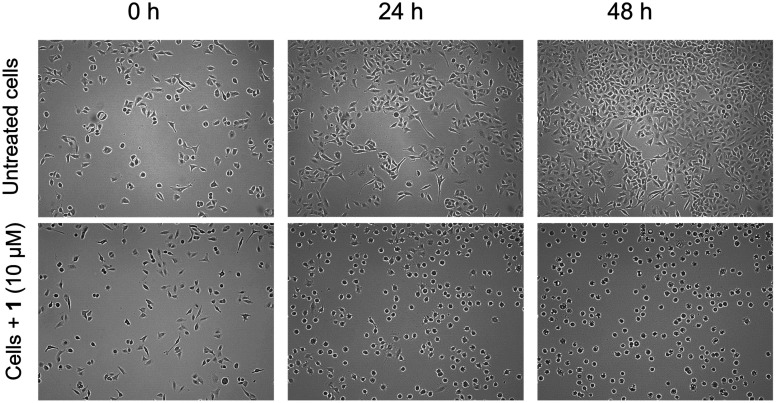
Live cell imaging in A549 cells with and without 1 (10 μM), showing changes in the cell morphology at 0, 24 and 48 h.

Live cell imaging was performed and microscopy images were recorded to monitor the morphological changes of A549 cells treated with 1 (Fig. 4). The cells were grown until at least 30% confluency before 10 μM 1 was added and images were recorded every hour for 48 h. The concentrations used for the experiments were based on the IC_50_ values of 1 in the respective cell line. Whereas untreated control cells showed a continuous extension of the cell layer leading to increased confluency, cells treated with 1 showed major morphological changes within the first 8–12 h of exposure. Cells were strongly deformed compared to the untreated control samples and rounded with longer exposure times. Cell growth continued, however, the cell morphology was substantially affected as evident from the shrunken cell morphology (Fig. 4). This effect was not reversible and cells completely detached over extended periods of exposure. This further corroborates the *in vitro* cytotoxic properties of 1. In our previous report on the analogous triphenylphosphine gold(i) alkyne complex, the morphology of RC-124 or HT-29 cells was affected only after longer exposure (48 h), followed by strong rounding and detachment of the cells.^32^ The shrunken cell morphology and rounding were also noted for a gold alkyne complex with an erlotinib-derived ligand.^26^

### 2.6 Wound healing assay

Metastasis is the primary cause of cancer morbidity and mortality and it is estimated that metastases are responsible for about 90% of cancer deaths. The wound-healing assay is a simple and convenient method to evaluate directional cell migration *in vitro*. Migration is a critical process involved in morphogenesis, inflammation, and cancer metastasis. Many metal complexes including those based on gold have been reported to show antimetastatic properties *in vitro*^33,45,46^ To check the antimetastatic properties of 1, we performed a wound healing assay using MDA-MB-231 cells, in which activity was reported with a different gold alkynyl complex already.^26^ The MDA-MB-231 cell line is known for its strong cell migration and is more suitable for the performed wound healing assay than the A549 cell line, as we also confirmed in pilot experiments.

**Fig. 5:**
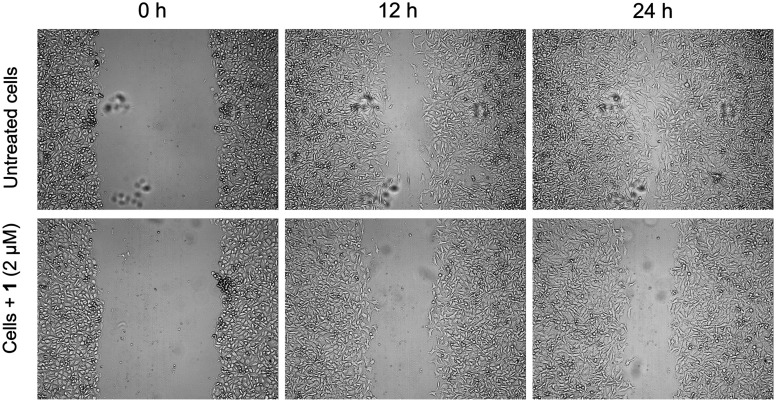
Effect of 1 on MDA-MB-231 cells after inducing a scratch and allowing the cells to grow for 24 h. Untreated cells showed nearly complete wound closure in 24 h while the cells treated with 1 (2 μM) showed a reduced rate of wound closure.

Cells were allowed to grow until they attained 60–70% confluency and treated with 1 at 2 μM. In a typical scratch wound healing assay, a “wound gap” in the cell monolayer was created by scratching, and the “healing” of this gap by cell migration and growth towards the center of the gap was monitored under a microscope for 48 h. Factors that alter the motility and/or growth of the cells can lead to an increased or a decreased rate of “healing” of the gap. Complex 1 was found to delay the rate of wound healing in the triple negative MDA-MB-231 breast cancer cell line compared to the untreated control cells, as demonstrated in Fig. 5. The *t*_1/2_ (time taken for closure of half the wound) was calculated to be 41.8 ± 3.3 h, while that for the untreated cells was 28.7 ± 2.7 h. This is indicative of the *in vitro* antimetastatic properties of complex 1.

### 2.7 Effect on mitochondrial respiration

**Fig. 6:**
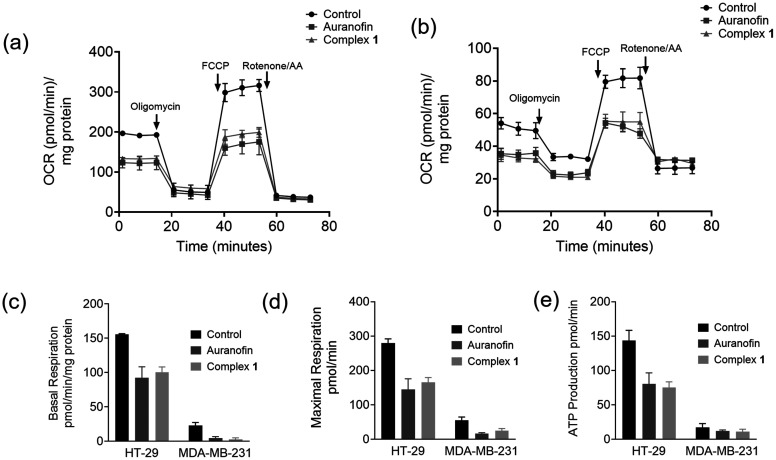
Seahorse Mito stress assay in HT-29 cells (a) and MDA-MB-231 cells (b) showing the effect of 1 and auranofin on mitochondrial respiration. Oligomycin (ATP synthase inhibitor), FCCP (proton decoupler), rotenone (electron transport chain complex 3 inhibitor) and rotenone (electron transport chain complex 1 inhibitor) were added at the time points shown by the arrows. Comparison of the respiratory parameters between untreated cells (control) and cells treated with either 1 or auranofin: basal respiration (c), maximal respiration (d) and ATP production (e) in HT-29 and MDA-MB-231 cells as indicated in the graphs.

Many gold complexes, including gold alkynyl derivatives, cause effects on mitochondrial metabolism.^18,45,46^ Here, we performed studies on the mitochondrial respiration that could be affected by 1 using an Agilent XFe96 Seahorse extracellular flux analyzer. The real time oxygen consumption rate (OCR) and extracellular acidification rate (ECAR) of treated cells were measured (Fig. 6). Studies on the effects of metal complexes on the metabolism of cancer cells using real time OCR measurements have been reported only recently.^51–53^ This prompted us to explore the effect of 1 and auranofin as a reference on the oxidative phosphorylation in HT-29 and MDA-MB-231 cells. Cells were treated with 5 μM of the test compounds and incubated for 24 h. Mitochondrial respiration was found to be severely impaired in cells treated with the two compounds, as opposed to the untreated cells. This was evident from the low basal respiration and the inhibition of ATP production compared to those of untreated cells. The mitochondrial membrane of the cells treated with 1 or auranofin lost the capacity to restore the proton balance when treated with an uncoupling agent (FCCP). The maximal respiration (the OCR value when the mitochondrial membrane is uncoupled) and spare respiratory capacity (difference in the OCR values between maximal respiration and basal respiration) of the cells were reduced compared to those of untreated cells. The combination of these effects suggests a disrupted mitochondrial respiration in colorectal cancer cells caused by both 1 and auranofin. Strong effects on cellular respiration were also noted for structural derivatives of 1, such as its triphenylphosphine analogue.^45,46^

### 2.8 Inhibition of thioredoxin reductase activity

The inhibition assay of TrxR was performed with the isolated rat liver enzyme in a 5,5′-dithiobis-(2-nitrobenzoic acid) (DTNB)-based assay, and the IC_50_ values were calculated after incubation for 75 min at 37 °C. Complex 1 was incubated with the enzyme at different concentrations and the conversion of DTNB to TNB was monitored at 405 nm spectrophotometrically. Complex 1 was a strong inhibitor of the enzyme with an IC_50_ value in the nanomolar range that was identical to the value obtained in our previous report.^32^

Having confirmed the strong potency of 1 as an inhibitor of purified TrxR, the study was extended to lung cancer A549 cells that are reported to overexpress thioredoxin reductase.^54^ To check the efficacy of intracellular enzyme inhibition by 1 in A549 cells, we established an end point insulin reduction assay in our group using a similar method reported by Holmgren *et al.* and Arner *et al.*^55–57^ Cells were incubated with either 1 or auranofin at different concentrations for 24 h. The cell lysates were then analyzed using an insulin reduction method, where the difference in absorption between samples with and without thioredoxin was used and the conversion of DTNB to TNB was monitored. With an IC_50_ value of approximately 0.5 μM, the cellular TrxR inhibition by 1 was found to be comparable to that of auranofin (Table 2). For both gold compounds, the IC_50_ values obtained with A549 cell lysates were found to be at least ten times higher than with the isolated enzyme. In the cell-based experiment, the effective free drug concentration can be reduced due to many factors, such as cellular uptake or protein binding. In consequence, the different “bioavailability” between the experiment using a purified enzyme and the cell-based assay leads to the differences in the observed IC_50_ values.

**Table 2:** Inhibition of purified or cellular thioredoxin reductase (TrxR) by 1 and auranofin (*n* = 2)

Compound	Extracellular TrxR (rat liver enzyme)	Intracellular TrxR (A549 cells)
IC_50_ values (μM)		
1 (4-MeO-Ph-≡-Au-PEt_3_)	0.05 ± 0.01	0.45 ± 0.02
Auranofin (AF)	0.03 ± 0.46	0.69 ± 0.16

**Fig. 7:**
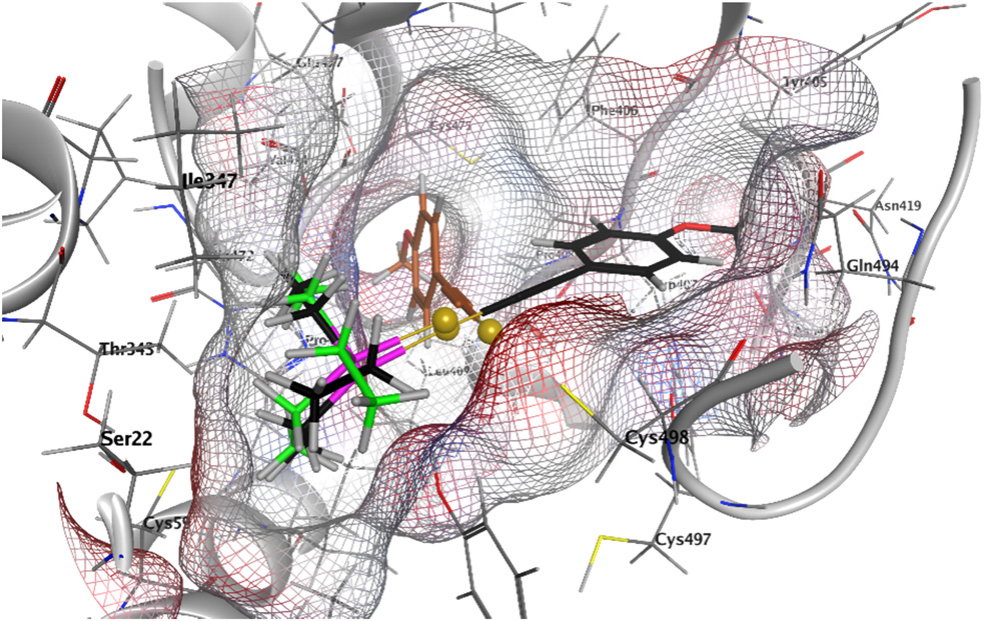
Docking of 1 (black), (phosphine)Au^+^ (green) and alkynyl-Au^+^ (brown) into TrxR1 (PDB: 2J3N, Sec → Cys); receptor surface: H-bonding (red), mild polar (blue), and hydrophobic (grey).

Docking studies were performed in order to obtain deeper insights into the molecular interactions of 1 with the TrxR binding site. Fragments of 1, *i.e.* alkynyl-Au^+^ and (phosphine)Au^+^, were included in the study to evaluate the effects of the respective partial structures. The docking results of complex 1 (black), alkynyl-Au^+^ (brown), and (phosphine)Au^+^ (green) with the human TrxR1 (PDB: 2J3N, Sec → Cys) reveal critical insights into their binding interactions, particularly in the vicinity of the active-site residue Cys498 (Fig. 7 and S1–S3†). The receptor surface grid, visualized in the docking study, highlights potential hydrogen bonding regions (red), mild polar regions (blue), and hydrophobic regions (grey), providing a detailed understanding of the ligand–receptor interface. Complex 1 demonstrates the strongest binding affinity with a score of −6.027, followed by alkynyl-Au^+^ (−4.176) and (phosphine)Au^+^ (−4.173). This suggests that 1 (Fig. 7 and S1†) forms the most stable interactions within the active site. The docking poses show that (phosphine)Au^+^ (Fig. 7 and S2†) occupies a similar position in the binding pocket and interacts with the key residues in a comparable manner. This similarity indicates that (phosphine)Au^+^ could in principle form through ligand exchange and a subsequent reaction of 1 with Cys498, which itself was navigated in the ideal position in the receptor through the alkynyl ligand and as a consequence of the high linearity of the structure of 1. On the other hand, the alkynyl-Au^+^ fragment itself can be positioned in different orientations, preferentially in a rectangular one, compared with 1 (Fig. 7, S2 and S3†). In summary, these mechanistic studies are in good agreement with the strong TrxR inhibition observed with 1 and indicate the special relevance of the phosphine ligand to the orientation of 1 in the TrxR binding site.

### 2.9 Toxicity study in a mouse xenograft model

Toxicity and tolerance studies were performed to evaluate the tolerability (MTD) of 1 at three different doses where female NMRI nu/nu mice were treated i.p. at doses of 5 (group A), 10 (group B) and 20 (group C) mg kg^−1^ for 5 days and the body weight was measured during this treatment. Furthermore, animal health conditions were monitored during the study. The MTD testing revealed in general good tolerability for both compounds, and throughout only minor and reversible losses in body weight were seen for the dose of 20 mg kg^−1^ of 1 (2.6%). At day 3 of the treatment, animals of groups A to C had slight diarrhea, which however was only transient. Furthermore, for group C, which received 20 mg kg^−1^1, liver degeneration was observed during necropsy after study termination. This MTD study showed good tolerability of 1 for i.p. application in the dose range between 5 and 10 mg kg^−1^. Therefore 10 mg kg^−1^ dose was used for further efficacy testing in an appropriate tumor model.

### 2.10 Tumor reduction study

**Fig. 8:**
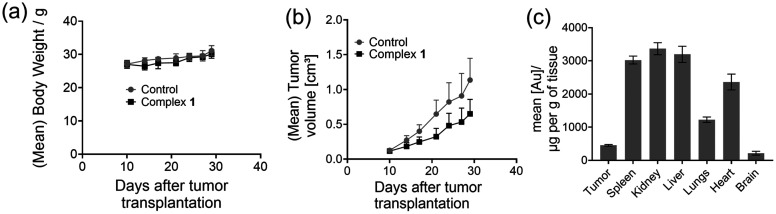
*In vivo* study in an A549 lung cancer xenograft model in animal groups treated with the vehicle or complex 1 (10 mg kg^−1^): mean body weight (a) and tumor volume (b). Biodistribution of gold in vital organs of mice treated with 1 (*n* = 5) (c).

This study was performed to evaluate the antitumoral activity of 1 in the A549 lung carcinoma CDX s.c. model. For testing of the compound, female NMRI nu/nu mice were injected s.c. A549 cells and tumours were grown to palpable size. Then, the animals were treated with 1 at a fixed dose of 10 mg kg^−1^ every second day by i.p. injections. The treatments were performed for 20 days for the two drugs. During the study, the tumour volume and animal body weight were determined. The study revealed antitumoral activity of 1 in the lung carcinoma CDX model A549 model. This is reflected by the respective opt. *T*/*C* values of 49.9% at study day 21 and by reduced tumour weights. In the control group, the mean tumor weight was 0.775 g, while in the group treated with 1, the mean tumour weight was 0.531 g (Fig. 8). The statistical analysis revealed that at study day 29, unpaired *t* tests with Welch's correction showed significant differences between the tumor volumes of the treatment groups “vehicle” and 1. During the entire study, treatments with 1 were well tolerated, which is reflected by no losses in animal body weights. This study demonstrated good antitumoral activity of 1 at a fixed dose of 10 mg kg^−1^.

The biodistribution of a drug molecule is an important pharmacokinetic feature, which plays an important role in determining the drug efficacy. To study the biodistribution of 1 in various vital organs, the organs from the animals in the tumor reduction study were collected after sacrificing the animals, weighed and digested with 10% nitric acid. Once the digestion was complete (3–5 days), the amount of gold in the different samples was analyzed using an atomic absorption spectrophotometer and the concentration of gold per gram of tissue was calculated. Significant amounts of gold were found in the liver, kidneys and spleen while a negligible amount was found in the brain (Fig. 8). Modest amounts of gold were found in the tumor and some in the other vital organs.

## 3. Discussion and conclusions

Gold complexes have been used to treat symptoms of rheumatoid arthritis for many decades and currently the registered therapeutic gold compounds are also considered as anticancer and anti-infective drug candidates. This has led to intensive research on novel gold containing small molecules in particular as anticancer agents. However, while many new *in vitro* active gold metallodrugs have been reported, extended preclinical studies, pharmaceutical analytical studies, and *in vivo* studies are less common. In this paper, we report on such extended studies with the previously reported gold organometallic lead compound 1. There are many reports on the anticancer activity of gold(i) and gold(iii) complexes, with thioredoxin reductase or similar oxidoreductase enzyme inhibition as the primary mechanism of action. Here, we have studied an organometallic gold(i)–phosphine complex having an alkynyl ligand with high stability under standard laboratory conditions. It showed excellent *in vitro* antiproliferative activity in different cancer cell lines with inhibitory concentrations similar to the FDA approved drug auranofin that is in clinical trials for anticancer and antimicrobial applications. Interaction studies with NAC confirmed the “thiol-reactivity” of 1 and the release of the metal free ethynylanisole ligand, which itself did not trigger significant cytotoxicity in the *in vitro* studies. The wound healing assay indicated that 1 might have additional antimetastatic properties. In addition, the complex was a prolific inhibitor of both purified and cellular TrxR. Docking studies further supported the experimentally confirmed strong TrxR inhibition by 1. Further, these studies indicated the special relevance of the phosphine ligand to the orientation of 1 in the active site and as a possible inhibitory active fragment in the form of (phosphine)Au^+^ (after the reaction with the cysteine in the active site). It can be speculated that the main role of the ethynylanisole ligand is to navigate and stabilize the (phosphine)Au^+^ fragment in a good position for this interaction.

Further experimental studies showed that 1 had a strong effect on mitochondrial respiration. Among manifold causes (*e.g.* the inhibition of ATP synthesis or the uncoupling of oxidative phosphorylation), the inhibition of TrxR can lead to an impairment of the mitochondrial respiration *via* oxidative stress and perturbed redox homeostasis.

Very importantly, we studied the *in vivo* efficacy of the molecule regarding the tolerability and tumor reduction in an A549 lung cancer xenograft model, where significant differences were observed between untreated and treated animals. Complex 1 with the triethylphosphine ligand presents a significant improvement compared with its triphenylphosphine precursor, which has shown no activity *in vivo* due to limitations in bioavailability.^32^ Further studies might reveal, if modification in the drug application schedule or a moderate increase in dosing could improve the antitumoral activity of these drugs. Furthermore, it will be of interest to evaluate if the metallodrug candidate is also effective in other models of different tumor entities. Notably, 1 was well tolerated *in vivo*, although no selective cytotoxicity has been observed in the *in vitro* cytotoxicity assays.

In summary, 1 represents a promising novel anticancer drug candidate and lead compound that is highly stable under physiological conditions, acts as a “thiol-reactive” agent and potent TrxR and mitochondrial respiration inhibitor, and has strong *in vivo* anticancer activity. This organometallic gold compound thus features properties different from those of the lead platinum metallodrug cisplatin, in particular regarding the mechanism of action (cisplatin: DNA binding, 1: TrxR inhibitor).

## 4. Materials and methods

### 4.1 General

Chemicals and reagents and solvents were procured from Sigma Aldrich and stored under recommended conditions. All reagents were used without further purification. Details of the instruments used are mentioned in the respective experimental sections.

#### Cell culture methods

MDA-MB-231, MCF-7, A549, and HT-29 cells were maintained in Dulbecco's modified Eagle medium (4.5 g L^−1^d-glucose, l-glutamine, pyruvate), which was supplemented with gentamycin (12.5 mg L^−1^) and fetal bovine serum (Biochrom GmbH, Berlin) (10% V/V), and were passaged once a week. RC-124 healthy human kidney cells were maintained in McCoy's 5A (modified, with l-glutamine) medium, which was supplemented with gentamycin (12.5 mg L^−1^) and fetal bovine serum (Biochrom GmbH, Berlin) (10% V/V), and were also passaged once a week. For experiments with RC-124 cells, microtiter plates have been pretreated in the following way: 30 μL of a sterilized gelatin solution (1.5% (m V^−1^)) were added to each well of flat bottom 96-well plates, the plates were covered with their lids and incubated for 1 h at 37 °C, the excess solution was removed, the wells were washed with PBS 7.4 pH, and the new cell culture medium was added. 175 cm^2^ cell culture flasks used for cultivation of RC-124 cells were pretreated analogously.

#### Instruments

The gold content for different experiments was measured using either an atomic absorption spectrophotometer (AAS; contrAA 700 high-resolution continuum-source atomic absorption spectrometer, Analytik Jena AG) or an inductively coupled plasma optical emission spectrophotometer (ICP-OES; Agilent Technologies ICP-OES, 5110 equipped with an SPS4 autosampler) as indicated in the respective experiments. For AAS measurements, pure samples of complex 1 were used to prepare the standard solutions and calibration was done in a matrix-matched manner. The mean integrated absorbance of triple injections was used for all the studies. Triton-X 100 (1%, 10 μL) and ascorbic acid (1%, 10 μL) were directly added to each standard sample (100 μL). Samples were injected (25 μL) into coated standard graphite tubes (Analytik Jena AG) and thermally processed, and gold was quantified at a wavelength of 242.7949 nm. For ICP-OES, standard solutions of gold were prepared using commercially available 1 g L^−1^ Au standard, ICP grade, from Sigma Aldrich, calibration was done in a matrix-matched manner and gold was quantified at 242.795 nm.

The mitochondrial respiration parameters (oxygen consumption rate (OCR)) were measured in real time using an Agilent Seahorse extracellular flux analyzer, and the values of basal respiration, maximal respiration and ATP production were calculated from the data obtained.

### 4.2 Synthesis and characterization

Complex 1 was synthesized by a method similar to that reported before with some modifications to improve the reaction yield.^32^ 1-Ethynyl-4-methoxybenzene (35.6 mg, 0.269 mmol) and KOH (90.50 mg,1.61 mmol) were dissolved in methanol (30 mL). Chloro(triethylphosphine)gold(i) (78.6 mg, 0.224 mmol) dissolved in dichloromethane (1.0 mL) was slowly added, and the solution was heated under reflux conditions for 6 h followed by stirring at room temperature for another 72 h. The solvents were removed by evaporation, the remaining residue was dissolved in dichloromethane and extracted thrice with water, the combined organic phases were dried over MgSO_4_ and filtered, and the solvents were removed by evaporation to afford a yellow powder (yield ∼80%). ^1^H NMR (CDCl_3_, Fig. S4†): 1.20 (m, 9 H, –CH_3_), 1.80 (m, 6 H, PCH_2_); 3.78 (s, 3 H, OCH_3_); 6.77 (m, 2H, ArH), 7.42 (m, 2H, ArH); ^13^C NMR (CDCl_3_, Fig. S5†): 8.92 (CH_3_), 17.86 (d, CH_2_, *J* = 33.0 Hz), 55.16 (OCH_3_), 113.57 (ArC), 117 (ArC), 133.67 (ArC), 158.41 (ArC), C≡C signals not observed; ^31^P-NMR (CDCl_3_, Fig. S6†): 38.65 (s); elemental analysis (found/theoretical): C (40.28/40.37); H (5.02/4.97).

### 4.3 Stability studies by HPLC-MS

Complex 1 was prepared as a stock solution in DMSO. *N*-Acetylcysteine (NAC) was prepared as a stock solution in PBS. For stability studies, the following samples were prepared: 400 μM 1 in DMSO and 400 μM 1 and 400 μM NAC in DMSO/PBS 1/1; the samples were kept in vials at 37 °C in a HPLC autosampler and were injected fully automatically after 0, 2, 4, 8, 12, 24, 48, 72 and 96 hours. For HPLC-MS analysis, an Agilent 1620 apparatus equipped with a single quadrupole mass spectrometer (Agilent 6120B) was used. The instrumental setup and chromatographic conditions are as follows: injection volume: 3.0 μL; flow rate: 0.8 mL min^−1^; autosampler temperature: 37 °C, column temperature: 40 °C; mobile phase: acetonitrile/ammonium formate buffer pH 4.0 (10 mM) with 0.02% formic acid 1/1; stationary phase: ACE UltraCore 2.5 SuperC18 (4.6 × 50 mm, particle size: 2.5 μm); detection wavelength: 290 nm. The identity of the compounds was confirmed by MS detection. Linear calibration with stock solutions of 1 afforded a correlation coefficient of 0.999.

### 4.4 Cell proliferation inhibition (crystal violet assay)

A number of 3000 cells per well were seeded in 96-well plates (note: for RC-124, pretreated plates were used, see above) and incubated at 37 °C in 5% CO_2_ for 24 h. Stock solutions of the compounds in dimethylformamide (DMF) were freshly prepared and diluted with the respective cell culture medium to graded concentrations (final concentration of DMF: 0.1% v/v). After 72 h (MDA-MB-231, MCF-7, A549, and HT-29) or 96 h (RC-124) of incubation, the cell biomass was determined by crystal violet staining using an X4 Victor Perkin Elmer plate reader at 595 nm, and the IC_50_ value was determined as the concentration that caused 50% inhibition of cell proliferation compared to an untreated control. Results were calculated as the mean of three independent experiments.

### 4.5 Live cell imaging

A number of 1 × 10^5^ A549 cells or HT-29 cells were grown in 60 mm cell culture dishes until at least 30% confluency. Complex 1 was prepared freshly as 10 mM stock solution in DMF and diluted to 10 μM with DMEM supplemented with 10% FBS. The cell culture medium of the flasks was replenished with a fresh medium containing the complex at the indicated concentration. Imaging was performed using a JuLI^Br^ live cell movie analyzer (NanoEnTek) equipped with two microscope units. In each experiment, one microscope unit was used to monitor an untreated control and the other microscope unit was used to monitor the cells treated with 1. The microscope units were placed in a CO_2_-incubator at 37 °C and images were taken in 1 h intervals for a period of 48 h. Each experiment was performed twice on separate days and afforded similar results.

### 4.6 Protein binding

The protein binding of the gold complex was studied using a precipitation assay similar to that reported earlier by Ma *et al.*^58^ Fetal bovine serum was chosen as the representative protein since it was used in all the cell culture experiments. The cell culture medium (DMEM) was reconstituted with 10% FBS. 1 was dissolved in DMF to make a stock solution (10 mM). It was added to the reconstituted medium such that the final concentration was 50 μM in a total volume of 5 mL. The samples were placed in an incubator at 37 °C for different periods (0, 2, 4, 8, 24 and 48 h). The untreated reconstituted cell culture medium was treated identically to the other samples and used as a matrix for calibration using a standard solution of gold (1.0 ppm Au standard solution procured from Sigma Aldrich, ICP grade). Aliquots (500 μL) of each sample were treated with ice-cold ethanol (1 mL) and stored at −25 °C for 3 h. Thereafter, the samples were centrifuged (964*g* at 4 °C for 20 min). The supernatant was isolated and stored at −25 °C for further measurements. Aliquots (500 μL) of each sample were treated with 10% nitric acid (4.5 mL), and the samples were further diluted using double distilled water. The gold concentration in the samples was measured using an inductively coupled plasma optical emission spectrometer (Agilent Technologies ICP-OES, 5110) equipped with an SPS4 autosampler. The values reported are the average of three independent measurements.

### 4.7 Cellular uptake

The cellular metal uptake was determined according to previously described methods.^23^ Briefly, MDA-MB-231 and A549 cells were grown until at least 75–80% confluency in 150 cm^2^ cell culture flasks. Stock solutions of the compounds in DMF were prepared and diluted with a cell culture medium immediately before use (final DMF concentration: 0.1% v/v). The cell culture medium of the flasks was replaced with a medium that contained the metal compound (20 mL), and the flasks were incubated at 37 °C in 5% CO_2_ for up to 48 h. After the desired incubation period, the uptake was stopped by removing the cell culture medium. The cells were washed with PBS (10 mL), the washing solution was removed, and the cells were isolated after 6 min trypsinization (2.4 mL trypsin solution 0.05%, containing EDTA 0.004%) by centrifugation (5 min, 1096*g*). The obtained cell pellets were stored at −20 °C for further use. For metal and protein quantification, the pellets were resuspended in demineralized water (1.0 mL) and lysed for 30 min by ultrasonication. The protein content of lysates was determined by the Bradford method, and the metal content was determined using a high-resolution continuum source atomic absorption spectrometer (ContrAA 700, AnalytikJena AG) using the matrix matched method.

### 4.8 Wound healing assay

The wound healing ability of complex 1 was evaluated in MDA-MB-231 triple negative breast cancer cells using a reported literature procedure.^59^ Briefly, 1 × 10^6^ cells were seeded in 60 mm cell culture dishes in DMEM having 10% FBS and allowed to grow for 24 h at 37 °C in a 5% CO_2_ atmosphere in an incubator. A scratch was made in the cell monolayer using a pipette tip (200 μL). The cells were washed 3 times to remove the floating cells and either 0.1% DMF or 1 (1.0 μM) was added to the cells. Images were recorded using a JuLI^Br^ live cell movie analyzer (NanoEnTek) equipped with two microscope units. The images were processed with ImageJ software where the area of the scratch was plotted against time to obtain the *t*_1/2_ value using a linear fit equation.

### 4.9 Seahorse Mito stress assay

HT-29 and MDA-MB-231 cells were seeded at a density of 20 000 cells per well in Seahorse XFe96 well plates in DMEM supplemented with 10% FBS, 1% sodium pyruvate and 1% HEPES buffer at 37 °C in 5% CO_2_. After 24 h, the medium was replaced with a fresh medium and either auranofin or complex 1 (1 μM) was added into the respective wells. After 24 h of incubation, the medium was removed and the cells were washed thrice using bicarbonate and serum free DMEM, supplemented with 1.8 mg mL^−1^d-glucose, 1% l-glutamine and 1% sodium pyruvate, and incubated in a non-CO_2_ incubator at 37 °C for 1 h. The Mito stress assay was run using oligomycin (1 μM), FCCP (1 μM), and a mixture of antimycin-A/rotenone (1 μM) each in ports A, B, and C, respectively, using a Seahorse XFe96 extracellular flux analyzer. The protein content of the samples was measured using the BCA assay.

### 4.10 Thioredoxin reductase inhibition (purified enzyme)

To determine the inhibition of mammalian thioredoxin reductase, a spectrophotometric assay was done using commercially available rat liver TrxR (Sigma-Aldrich) as reported previously.^32^ Briefly, the enzyme was diluted with distilled water to achieve a concentration of 2.5 U mL^−1^. Complex 1 was freshly dissolved in DMF to obtain a stock solution of 10 mM. To a 25 μL aliquot of the enzyme solution, 25 μL of potassium phosphate buffer, pH 7.0, containing the complex at different concentrations or the vehicle (DMF) without compounds (control probe), was added, and the resulting solutions (final concentration of DMF: max. 0.5% v/v) were incubated with moderate shaking for 75 min at 37 °C in a 96-well plate. Subsequently, to each well, 225 μL of reaction mixture (1000 μL of reaction mixture consisting of 500 μL of potassium phosphate buffer, pH 7.0, 80 μL of EDTA solution (100 mM, pH 7.5), 20 μL of BSA solution (0.2%), 100 μL of NADPH solution (20 mM), and 300 μL of distilled water) was added. The reaction was started by the addition of 25 μL of an ethanolic 5,5′-dithiobis 2-nitrobenzoic acid solution (DTNB, 20 mM). After proper mixing, the formation of 5-TNB was monitored with a microplate reader (Perkin-Elmer Victor X4) at 405 nm in 10 s intervals for 10 min. The increase in 5-TNB concentration over time followed a linear trend (*r*^2^ ≥ 0.99), and the enzymatic activities were calculated as the slopes (increase in absorbance per second) thereof. For each tested compound, the noninterference with the assay components was confirmed by a negative control experiment using an enzyme free solution. The IC_50_ values were calculated as the concentration of complexes decreasing the enzymatic activity of the untreated control by 50% and are given as the means and standard deviations of 2 independent experiments.

### 4.11 Thioredoxin reductase inhibition (A549 cells, endpoint insulin reduction assay)

The thioredoxin reductase inhibition assay was performed according to a method reported earlier.^55–57^ Briefly, A549 cells were seeded in 6-well plates at a density of 1 × 10^6^ cells per well in DMEM/10% FBS. After 24 h, they were treated with varied concentrations of the compounds dissolved in DMF (final DMF concentration was 0.1% v/v) and incubated at 37 °C in 5% CO_2_ for 24 h. Following this, the medium was removed, and the cells were washed twice with PBS and lysed with ice-cold lysis buffer (50 mM phosphate buffer, pH 7.4; 1 mM EDTA, 0.1% Triton-X 100) for 30 min on ice. The protein content in the samples was determined using the Bradford assay with bovine serum albumin (BSA) as the standard. Equal amounts of protein (10 μg) were used for all samples. Samples were incubated with the reaction mixture (30 μL) containing HEPES buffer (85 mM), insulin (0.3 mM), NADPH (660 μL), EDTA (3 mM), and either recombinant thioredoxin (10 μL, 1 mg ml^−1^) or HEPES buffer (10 μL) for 20 min at 37 °C. The reaction was stopped with a stopping solution (200 μL) containing guanidine hydrochloride and 5,5′-dithio-bis(2-nitrobenzoic acid) (DTNB). The absorbance was measured at 405 nm using a Perkin Elmer Victor X4 plate reader. The difference in the absorbance of samples containing Trx and buffer gives the activity due to thioredoxin reductase. The data were analyzed and the IC_50_ values were determined using a non-linear curve fit with Origin 8.0.

### 4.12 Computational chemistry (docking studies with TrxR)

In this study, computational analyses focused on the human thioredoxin reductase 1 (TrxR1). The protocol for protein preparation and pharmacophore docking was adapted from a previously published method with modifications to suit our experimental objectives.^28^ All computational work was performed using the Molecular Operating Environment (MOE, version 2024.0601). The X-ray crystal structure of TrxR1 (PDB Code: 2J3N) was retrieved from the Protein Data Bank (https://www.rcsb.org). Protein preparation involved removing bound water molecules and co-crystallized ligands, as well as correcting structural inconsistencies. Protonation states were assigned based on physiological conditions (temperature: 310.15 K, pH: 7.4, salt concentration: 0.9 M). Partial charges and hydrogen atoms were added to complete the structure. Notably, the cysteine residue Cys498, which is critical for ligand binding, was deprotonated and negative charge was assigned at the sulfur atom to reflect the active roles in catalysis as shown by Fritz-Wolf *et al.*^60^ Given the challenges associated with parameterizing metal complexes for conventional force fields, pharmacophore-based docking was employed to facilitate ligand placement. The binding site of TrxR1 was defined around the negatively charged sulfur atom of Cys498. Pharmacophore features with cationic characteristics were positioned in proximity and marked as essential to this residue to enable interactions with potential ligands. Complex 1 and ligand fragments, specifically alkynyl-Au^+^ and (phosphine)Au^+^, were constructed using the MOE. All structures were energy-minimized and saved in a single molecular database (.mdb) file. Similarly, the gold–phosphine fragment was modeled with a covalent bond between phosphorus and gold atoms, maintaining a tetrahedral geometry around phosphorus. Conformational searches were conducted for all structures using default MOE settings. The prepared ligand conformers were docked into the defined site of TrxR1 using an induced-fit docking protocol. Refinement of docked poses was performed using the Amber:EHT force field, while scoring employed the GBVI/WSA function to evaluate binding affinities.

### 4.13 *In vivo* studies

The animal studies were performed by EPO Experimental Pharmacology & Oncology (Berlin – Buch). For the studies, 6–8-week-old female NMRI nu/nu mice were used. In the first animal experiment, the maximum tolerable dose (MTD) of complex 1 was determined in female NMRI:nu/nu mice. 1 was dissolved in dimethylsulphoxide (DMSO) (final concentration 10%) and further diluted with 5% Tween 80 in saline. The mice (*n* = 2 mice per group) were administered with 5, 10 and 20 mg kg^−1^ of the complex intraperitoneally (i.p.) in daily injections of 0.1 ml for 5 days and further observed post treatment for 10 days in order to determine the approximate MTD. In this experiment, the body weight was measured and potential side effects were documented.

For the therapeutic *in vivo* experiment, 1 × 10^7^ A549 human lung carcinoma cells (expanded *in vitro* in RPMI medium + 10% fetal bovine serum) were injected subcutaneously (s.c.) in a volume of 0.1 mL into female NMRI:nu/nu mice (*n* = 5 mice per group) on day 0. When tumors were grown to a palpable size of around 0.1 cm^3^, the mice were randomized and treatment was started on day 10 after tumor cell injection. For treatment, complex 1 was injected i.p. at a dose of 10 mg kg^−1^, every second day for 20 days during the experiment. The control group of mice was treated with the solvent only. Tumor volumes (TVs) were measured with a caliper instrument at indicated time points and calculated using the formula: TV = (width^2^ × length)/2. Tumor volumes, relative tumor volumes (in relation to the first treatment day) and treated to control (*T*/*C*) values were calculated. The body weight and health conditions of the mice were determined continuously during the entire experiment to estimate tolerability of the drug treatment. The mice were sacrificed on study day 29 after their last treatment, and necropsy was performed for evaluation of potential side effects. Furthermore, tumors and organs (liver, lungs, kidneys, spleen, heart, brain) were removed and tumor weights were determined. The animal experiments were performed according to the German Animal Protection Law and with approval from the responsible local authorities (LaGeSo Berlin, Germany). Housing and animal care were in accordance with all legal and ethical regulations. The *in vivo* procedures for handling and treatment of mice were consistent and in compliance with the United Kingdom Co-ordinating Committee on Cancer Research (UKCCCR) guidelines. At the end of the study, animals were sacrificed and their vital organs were isolated, weighed and dissolved in 10% nitric acid for digestion. The gold content in the organs was measured using a high-resolution continuum source atomic absorption spectrometer (ContrAA 700, AnalytikJena AG) using the matrix matched method.

## Conflicts of interest

There are no conflicts of interest to declare.

## Supplementary Material

MD-016-D4MD00964A-s001

## Data Availability

The data supporting this article have been included as part of the manuscript and the ESI.†
