# Supplementary material for: Antiproliferative effects, mechanism of action and tumor reduction studies in a lung cancer xenograft mouse model of an organometallic gold(i) alkynyl complex
Source: RSC Med Chem. 2025 Mar 24;16(6):2663–76. doi: 10.1039/d4md00964a (PMC11975047; doi:10.1039/d4md00964a)
Supplement: MD-016-D4MD00964A-s001 [file MD-016-D4MD00964A-s001.pdf]

Supplementary Information (SI) for RSC Medicinal Chemistry.
This journal is © The Royal Society of Chemistry 2025

Supporting Information

Antiproliferative effects, Mechanism of Action and Tumor Reduction Studies in a Lung Cancer Xenograft Mouse Model of an Organometallic Gold(I) Alkynyl Complex

Uttara Basu *[a,b], Anna Wilsmann[a], Sebastian Türck[a], Henrik Hoffmeister[a], Matthias Schiedel[a], Gilles Gasser [c], Ingo Ott *, [a]

[a] *Institute of Medicinal and Pharmaceutical Chemistry, Technische Universität Braunschweig, Beethovenstr. 55, 38106 Braunschweig, Germany.*

[b] *Department of Chemistry, BITS Pilani K K Birla Goa Campus.*

[c] *Chimie ParisTech, PSL University, CNRS, Institute of Chemistry for Life and Health Sciences, F-75005 Paris, France.*

Table of contents

1) Figures S1 to S3; additional figures from the docking studies

2) Figures S4 to S6: NMR data

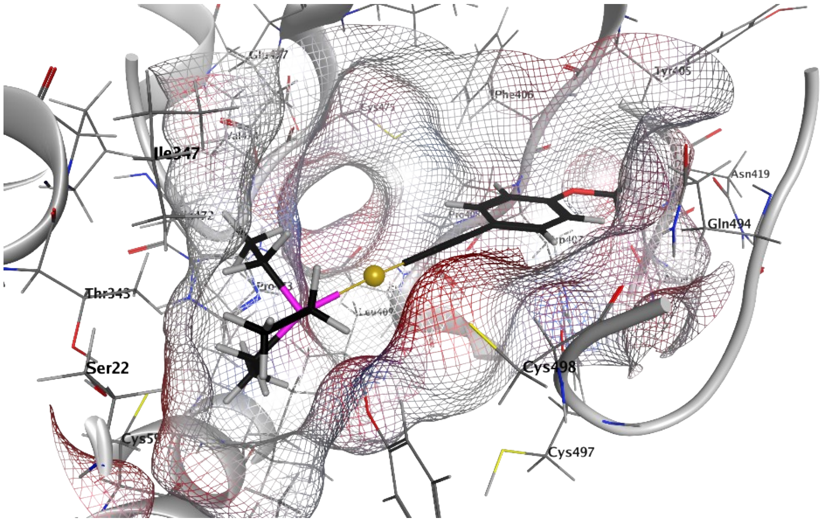

Figure S1: Docking of **1** into TrxR1 (PDB: 2J3N, Sec→Cys); receptor surface: H-bonding (red), mild polar (blue), hydrophobic (grey).

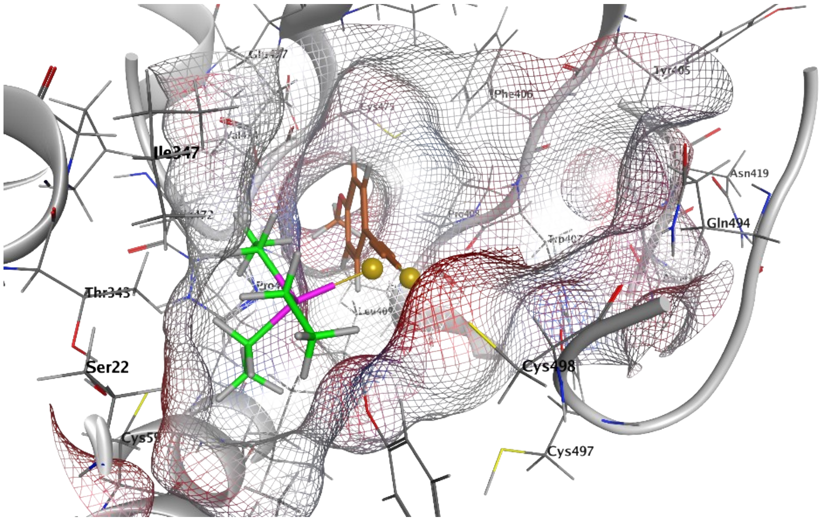

Figure S2: Docking of (phosphane)Au⁺ (green) and alkynyl-Au⁺ (brown) into TrxR1 (PDB: 2J3N, Sec→Cys); receptor surface: H-bonding (red), mild polar (blue), hydrophobic (grey)

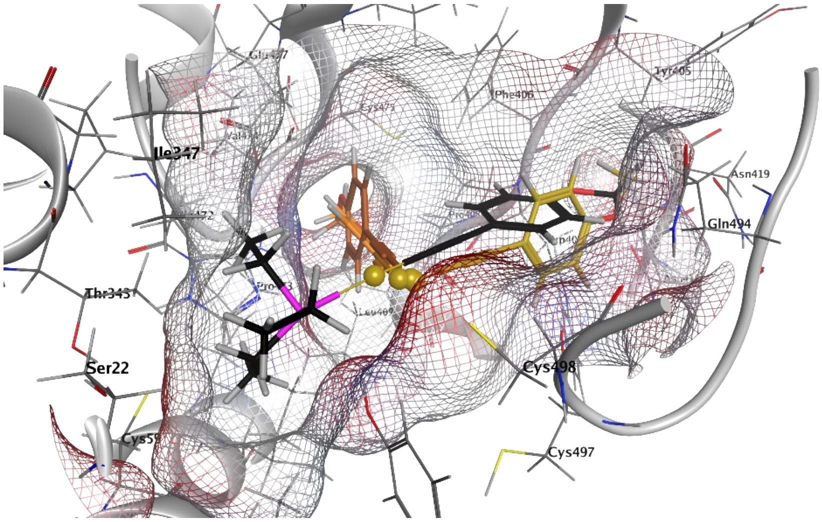

Figure S3: Docking of **1** and alkynyl-Au⁺ (brown, orange, yellow) into TrxR1 (PDB: 2J3N, Sec→Cys); receptor surface: H-bonding (red), mild polar (blue), hydrophobic (grey)

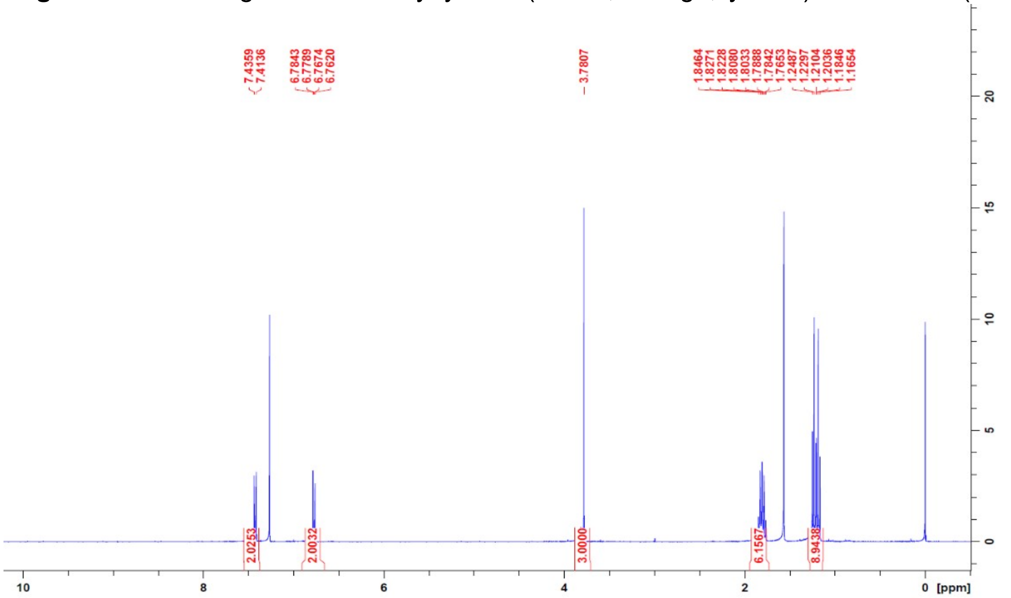

Figure S4: ^{1}H NMR of complex **1** recorded in $CDCl_3$

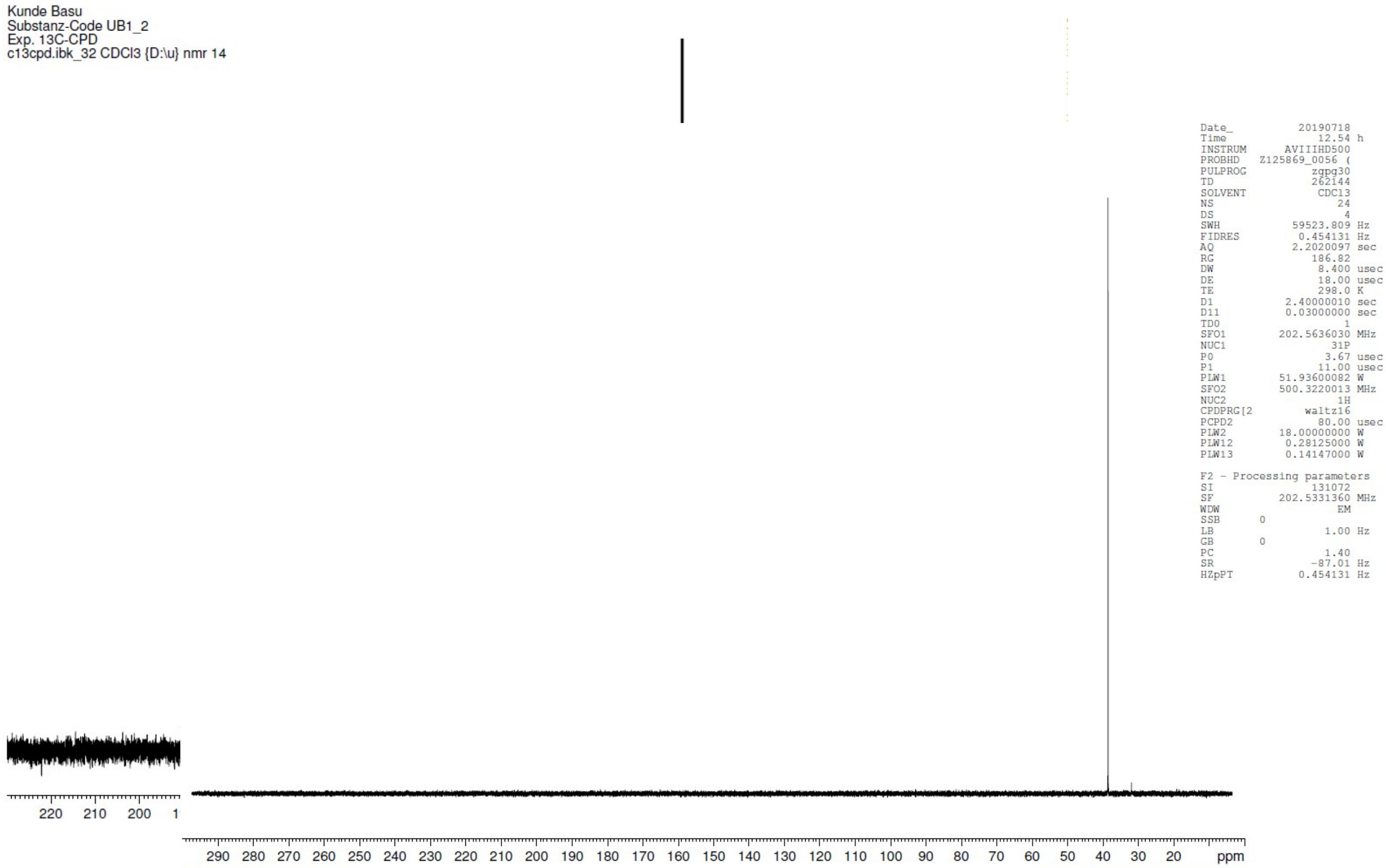

Figure S5: ^{13}C NMR of complex 1 recorded in $CDCl_3$

Figure S6: ^{31}P NMR of complex **1** recorded in $CDCl_3$
